# HMM-Based Action Recognition System for Elderly Healthcare by Colorizing Depth Map

**DOI:** 10.3390/ijerph191912055

**Published:** 2022-09-23

**Authors:** Ye Htet, Thi Thi Zin, Pyke Tin, Hiroki Tamura, Kazuhiro Kondo, Etsuo Chosa

**Affiliations:** 1 Interdisciplinary Graduate School of Agriculture and Engineering, University of Miyazaki, Miyazaki 889-2192, Japan; 2 Graduate School of Engineering, University of Miyazaki, Miyazaki 889-2192, Japan; 3 International Relation Center, University of Miyazaki, Miyazaki 889-2192, Japan; 4 Faculty of Medicine, University of Miyazaki, Miyazaki 889-1692, Japan

**Keywords:** action recognition, depth colorization, e-Healthcare, Hidden Markov Model, Histogram of Oriented Gradients, older persons, person detection, Support Vector Machine, Viterbi Algorithm, YOLOv5

## Abstract

Addressing the problems facing the elderly, whether living independently or in managed care facilities, is considered one of the most important applications for action recognition research. However, existing systems are not ready for automation, or for effective use in continuous operation. Therefore, we have developed theoretical and practical foundations for a new real-time action recognition system. This system is based on Hidden Markov Model (HMM) along with colorizing depth maps. The use of depth cameras provides privacy protection. Colorizing depth images in the hue color space enables compressing and visualizing depth data, and detecting persons. The specific detector used for person detection is You Look Only Once (YOLOv5). Appearance and motion features are extracted from depth map sequences and are represented with a Histogram of Oriented Gradients (HOG). These HOG feature vectors are transformed as the observation sequences and then fed into the HMM. Finally, the Viterbi Algorithm is applied to recognize the sequential actions. This system has been tested on real-world data featuring three participants in a care center. We tried out three combinations of HMM with classification algorithms and found that a fusion with Support Vector Machine (SVM) had the best average results, achieving an accuracy rate (84.04%).

## 1. Introduction

A huge issue of global importance is our aging population. Every country is facing a rapid increase in the number and ratio of older people in their population. Improved medical technology is one of the drivers of this phenomenon, as are falling fertility rates, increasing life expectancy at birth, and improved survival rates among the elderly. The United Nations (UN) regularly publishes estimates and projections for the world’s population. According to the 2019 Revision of its World Population Prospects [1], one in six people (16%) will be over age 65 by 2050, up from one in eleven (9%) in 2019. As another interesting fact, persons aged 65 or over outnumbered children under five for the first time in history, according to a report in 2018 [2]. Hence, working together to create a more age-friendly world is an urgent necessity. The global community should take specific actions for improving health and well-being into old age, and for developing supportive environments with varying degrees of independence.

With the current rates of aging in the population, the health of older adults is an increasing concern. Both physical and mental decline affect the quality of life enormously, as do unexpected falls. Because of low fertility rates in recent decades, most older people have only a couple of family members for support, or none at all. They usually go into care centers or live alone in their homes. Care centers play a vital role in ensuring that older people can live safely. Many older people reach a point at which they cannot live on their own. Everyone wants and should have the chance to live a long and healthy life. Luckily, we now have numerous assistive technologies. We have opportunities to build various types of systems for mitigating the hazards associated with growing older.

The objective of our proposed system is to provide quality long-term care. Our system not only helps prevent difficulties and accidents, but also enables older persons to live independently. In addition, the system can reduce the workload for caregivers who cannot monitor the elderly in person for 24 h a day. The various systems and applications developed to assist the elderly each take a different approach. We focused on recognizing daily activities performed by older people at a care center. For collecting data, we set up cameras for monitoring inside the rooms of the care center. We also need to incorporate feedback from the residents in developing a design that matched their needs [3]. This strategy engendered trust, and some residents volunteered to participate in our experiment. The participating residents in the care center are patients with both physical and mental impairments. We recorded their daily actions and activities in their rooms.

Our proposed system recognizes five basic actions: ‘Seated in the wheelchair’, ‘Standing’, ‘Sitting’, ‘Lying’, and ‘Transition’. The last action is important as it can pose a danger for older people. The risk is greatest during the transition from sitting to standing, standing to sitting, sitting to lying, and lying to sitting. Even though the other actions such as ‘taking medicine’ and ‘doing exercises’ are also important to investigate, the proposed system focuses on the above-mentioned five actions. These actions are performed by the participants at the care center on a daily basis when no nurses are present in their rooms to provide assistance. The system is expected to provide insight to both caregivers and those cared for by analyzing the results of the action recognition system.

The main contributions of the paper are as follows:

(1) To show how depth maps can be pre-processed by HUE colorization to detect persons before recognizing actions,
(2) To show how using the Hidden Markov Model (HMM) can effectively recognize the actions in continuous operations in real-time,
(3) To show how HMM can be combined with other classification algorithms to improve the recognition accuracy rate and,
(4) To show how the proposed system can automatically assist both the elderly and their caregivers, preserving the privacy of the users by using the depth camera.

The rest of the paper is organized as follows. Section 2 reviews literature related to the proposed system, Section 3 describes the overall proposed system, Section 4 presents experimental results, Section 5 provides some discussion, and Section 6 concludes the paper, including considerations for future research.

## 2. Related Works

This section provides a literature review concerning various approaches to action recognition for the elderly. Section 2.1 covers existing systems. Since our system is based on remote cameras for action recognition, privacy is very important. In Section 2.2, we review some notable works related to vision sensor-based action recognition, including the use of depth cameras for protecting privacy. Section 2.3 describes the previous contributions to image colorization and person detection. Finally, Section 2.4 describes related research into action recognition systems using HMM.

### 2.1. Elderly Health Supporting Technologies

On the subject of aging, new technologies in Artificial Intelligence (AI), such as Machine Learning (ML) and Deep Learning (DL), offer a broad range of opportunities [4]. Modern assistive technologies now include Ambient Assisted Living (AAL) systems, lifelogging technologies, gerontechnology, and smart homes, and are transforming many aspects of elder care [5]. The assistive technologies most applicable for older adults are types of ambient and mobile systems or services that improve or support the various functions, so that older people can continue leading healthy and independent lives [6]. Researchers in industry and academia have built numerous systems for older people based on wearable sensors (accelerometers and gyroscopic sensors) [7], ambient sensors (motion, radar, object pressure, and floor vibration sensors) [8], and vision sensors [9]. Some of the most useful systems include prompting and warning systems (reminder of daily routines), health monitoring (health analysis using portable activity sensors), support for people with dementia (detection of abnormal sleeping habits), and recognition of daily activities (use of cameras to detect and analyze activities). To maximize the comfort of those who are living in the care center, our proposed system relies on vision sensors (cameras) rather than wearable sensors to recognize actions.

### 2.2. Vision Sensor-Based Recognition Systems

Combined with the convenience of automatically inputting video sequences, action recognition systems that incorporate computer vision techniques with image processing technologies play a significant role in understanding what a subject is doing, and have consequentially become an active focus of research in recent years. For instance, the following studies were conducted on human action recognition using various types of vision sensors. The authors in [9] designed a monitoring and action recognition system by exploiting modern image processing techniques and RGB cameras. They trained the detection model in their system using the Faster Regions with Convolutional Neural Network features (R-CNN) by focusing on the ‘person’ class to locate the person. Again, the action recognition model was trained by the integration of Two-Stream Inflated 3D ConvNet (I3D) and Deep Human Action Recognition (DeepHAR) models. The authors introduced a new dataset with a large number of samples to balance the action samples, and designed a client-side, web-app interface for monitoring people. Another study [10] emphasized a method for real-time human action classification using a single RGB camera, which can also be integrated into a mobile robot platform. To extract skeletal joints from RGB data, the authors combined OpenPose and 3D-baseline libraries, then used Convolutional Neural Network (CNN) to identify the activities.

As more and more technologies emerge to assist older adults, researchers should consider the effect of health-related technologies on the people being monitored [11]. Most people want to keep their health information private, and they also worry about how such information could be used against them. According to surveys collected by the authors in [11], older adults have positive opinions of assistive technologies, but rarely accept systems that use cameras because of privacy concerns. To overcome this attitude, the use of depth cameras became more common for their advantages from a privacy perspective. By measuring distances between the camera and objects, depth data can be used for action recognition without using images that could be used to identify individuals. Furthermore, depth cameras can be used at the night without needing additional light. By using color with depth data, some authors have proposed a cloud-based approach [12] that recognizes human activities without compromising privacy. In this approach, researchers collect one motion-history image generated from color data, three depth-motion maps extracted from depth data, and then use deep CNN for the recognition process. Likewise, we also used a depth camera in our previous work [13], which introduced a real-time action recognition system that helps prevent accidents and supports the well-being of residents in care centers. In [13], we extracted both appearance-based depth features and distance-based features, extending the system described in [14] to recognize actions using the automatic rounding method. As another approach, [15] proposed a skeleton-based system for recognizing human activities for monitoring the elderly. They used Minkowski and cosine distances between 3D joint features for the recognition process, by characterizing the Spatio-Temporal components of a human activity sequence. The authors of [16] used 3D point clouds for action recognition by only processing depth maps. They developed a descriptor based on the Histogram of Oriented Principal Components for 3D action recognition (HOPC). The researchers used this descriptor to determine the Spatio-Temporal Key-Points (STKPs) in 3D point cloud sequences. In contrast to previous studies, our method relies on the depth map features of a stereo depth camera, and actions are recognized based on these features.

### 2.3. Depth Colorization and Object Detection

Among the various vision sensor-based action recognition systems, those using depth cameras have become more common than those using RGB cameras; as described in Section 2.2, they provide better privacy protection and can be used at night. On the other hand, the main drawback of using depth cameras is that they require a large amount of storage space for the depth data. Although numerous state-of-the-art codecs can provide high compression ratios for RGB image compression [17,18], those for depth images do not perform as well. By using colorization, depth images can be compressed to reduce storage space requirements, while still processing them as normal color images for visualization [19]. Our study investigated a system that colorizes depth maps, producing color images for the sake of compression, visualization, and object detection.

Before recognizing the actions of a person, person detection is an essential first step. Applications of deep learning and neural networks are becoming more sophisticated, and their use in deep learning-based models for object detection has increased substantially. Moreover, such object detection systems are increasingly used in real-world applications, such as autonomous driving, robot vision, and video surveillance [20]. Popular object detectors include Regions with CNN features (RCNN) [21] and its variants [22,23], You Only Look Once (YOLO) [24] and its variants [25,26,27,28], as well as Single Shot MultiBox Detector (SSD) [29]. Of these, recent versions of the YOLO object detector are more popular because of their significant advantages over other systems. This is especially true of the latest version of YOLO (YOLOv5 [28]), which is easy to train and use for inferencing. Two other works cited here used YOLOv5 as an object detector in their systems. The authors in [30] proposed a face-mask detection system using YOLOv5 to determine whether the person is wearing a face mask. Another used YOLOv5 to detect safety helmets in the workplace [31]. Due to its speed, YOLOv5 was used as a person detector in our system by focusing on the ‘person’ class.

### 2.4. HMM for Action Recognition

HMM is an extension of the Markov process which includes both hidden and visible states. It is a stochastic model, and is very rich in mathematical structure, while very useful for the sequential data encountered in practical applications [32]. HMM has found wide application in detection and recognition systems. Two common applications of HMM are in systems for fall detection and action or activity recognition. These systems are related, but have different objectives and use different types of data (collected using sensors or cameras). For a system using sensor data, the authors in [33] proposed a two-stage continuous HMM approach to recognizing human activities from temporal streams of sensory data (collected by accelerometer and gyroscope on a smartphone). The first level of HMM separated stationary and moving activities, while the second level separated data into their corresponding activity classes. Likewise, other research [34] has employed a two-layer HMM to build an activity recognition model using sensor data, but that differs from the model in the previous work [33]. In the first layer, location information obtained from the sensors was used to classify activity groups; in the second layer, individual activities in each group were classified. Then, they applied the Viterbi algorithm to their HMM to infer the activities. The activity recognition model in [35] established a Hierarchical HMM to detect ongoing activity by monitoring a live stream of sensor events. Their method also included two phases, but only the first phase used HMM. In this method, data streams were segmented according to the start and end points of activity patterns.

For systems relying on vision sensor data, the studies in [36,37] proposed HMM-based automatic fall detection systems with image processing techniques by utilizing RGB and RGB-D cameras, respectively. In [36], HMM was used as a decision-making process for differentiating abnormal (falling) from normal sequential states for a given person. The system made this decision by observing the six possible feature values which were defined according to the distance between the centroid of the person’s silhouette and the associated virtual ground point (VGP), the shape’s area, and the person’s aspect ratio. The HMM model was then developed by defining feature thresholds and calculating emission probabilities. On the other hand, [37] created an HMM model to detect and distinguish falling events from the other eight activities of the person. The observation symbols of their model were the vertical position of the center of mass, the vertical speed, and the standard deviation of all the points belonging to the person. In another study [38], a continuous HMM was used for human action recognition from the image data. The authors explicitly modeled the HMM using a temporal correlation between human postures, described using a Histogram of Oriented Gradients (HOG) for shape encoding, and a Histogram of Optical Flow (HOF) for motion encoding. Their HMM made continuous observations, modeling the probability distribution in each state by a mixture of Gaussians. Their experimental results showed that the continuous HMM outperformed recognition systems using a Support Vector Machine (SVM) based on Spatio-Temporal Interest Points (STIPs). In another study [39], an HMM was developed for a human activity recognition system in which the discrete symbols for HMM were generated by mapping into code words from estimated body joint-angle features. The HMM was trained for each activity, and the activities were then recognized using the trained models. Meanwhile, the authors in [40] and [41] had driven the development of Fisherposes for view-invariant action recognition using 3D skeleton data collected using a Kinect sensor. In [40], an HMM was used to characterize the temporal transition between body states in each action, and in [41], an HMM was used to classify actions in relation to an input series of poses. As for our system, we extended our prior work [42], which employed SVM for the action recognition process. In the current work, we developed an HMM model for elderly action recognition that uses space-time features to obtain observation symbols. Furthermore, we compared the results of various models which combine HMM with other classification models.

## 3. Proposed System

**Figure 1:**
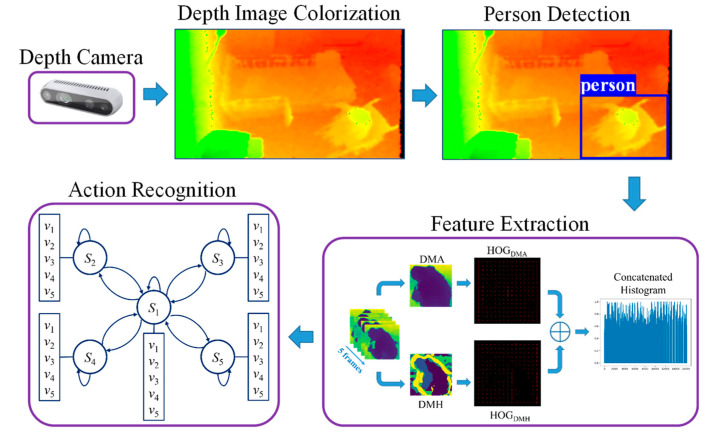
Overview of the proposed system.

The proposed action recognition system is composed of four processing subsystems: (i) depth data processing; (ii) person detection; (iii) feature extraction, and (iv) action recognition. Depth cameras are installed at an elderly care center to record the daily actions of three senior residents in three separate rooms. An overview of the process components and methods is shown in Figure 1.

Depth data processing is conducted in two parts. Firstly, the depth camera collects data. Secondly, depth image colorization is used to convert depth maps into color images. To recognize the actions of a person, that person must be detected within the camera’s field of view. This is conducted by the person detector for detecting the person’s bounding boxes in the colorized images. After the person is detected, the features are extracted from the person’s bounding boxes, and finally, the action recognition unit is performed using the HMM. The following subsections provide details for each process in these subsystems.

### 3.1. Depth Image Colorization

In the proposed system, depth image colorization is mainly used for compression, visualization, and person detection. In the case of compression, the depth cameras we used provided depth data with 16-bit depth resolution, which require a large amount of storage space for collecting and storing depth values in Comma Separated Values (CSV) format. By using colorization, depth data are compressed into RGB images, which reduce storage space requirements. Table 1 compares the storage space required for CSV files and compressed RGB images. For this comparison, one hour of video depth data is used. This hour-long, depth-data video is recorded at 1fps, thus containing 3600 depth frames. The CSV files store these depth frames as floating-point values, while the colorized files store the depth frames as color images. Hence, this is a comparison between the file sizes of 3600 CSV files and 3600 image files. According to the results, the CSV files require 17 times the storage space required for the compressed images.

**Figure 2:**
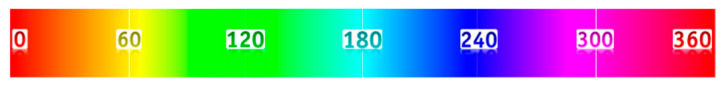
The hue color scale used for the colorization process.

As shown in Figure 2, hue color space is used for the colorization process. This color space has six scales in both directions of RGB channels and can thus be denoted as having 1529 discrete ranks, or approximately 10.5 bits [19]. Moreover, as one of the colors in the hue color space is always 255, the colorized images will not be too dark. This facilitated visualization, but also enable the use of the input images for the person detection process. Instead of the original depth values, we use an inverse colorization method, considered ‘inverse’ because of the disparity values, which are the reciprocals of depth values. We apply inverse colorization (1) to (5) from the whitepaper [19].

(1) $$disp=\frac{1}{d},\;\;\;\;\;disp_{max}=\frac{1}{d_{min}},\;\;\;\;\;disp_{min}=\frac{1}{d_{max}}$$

(2) $$d_{normal}=\frac{disp-disp_{min}}{disp_{max}-disp_{min}}*1529$$

(3) $$p_{r}=\left\{\begin{array}{ll} 255, & \;\mathrm{if}\;0\leq d_{normal}\leq 255\cup d_{normal}>1275\\ 255-d_{normal}, & \;\mathrm{if}\;255<d_{normal}\leq 510\\ 0, & \;\mathrm{if}\;510<d_{normal}\leq 1020\\ d_{normal}-1020, & \;\mathrm{if}\;1020<d_{normal}\leq 1275 \end{array}\right.$$

(4) $$p_{g}=\left\{\begin{array}{ll} d_{normal}, & \;\mathrm{if}\;0\leq d_{normal}\leq 255\\ 255, & \;\mathrm{if}\;255<d_{normal}\leq 510\\ 765-d_{normal}, & \;\mathrm{if}\;510<d_{normal}\leq 765\\ 0, & \;\mathrm{if}\;765<d_{normal}\leq 1529 \end{array}\right.$$

(5) $$p_{b}=\left\{\begin{array}{ll} 0, & \;\mathrm{if}\;0\leq d_{normal}\leq 765\\ d_{normal}-765, & \;\mathrm{if}\;765<d_{normal}\leq 1020\\ 255, & \;\mathrm{if}\;1020<d_{normal}\leq 1275\\ 1275-d_{normal}, & \;\mathrm{if}\;d_{normal}>1275 \end{array}\right.$$

where *d* is the depth value, *disp* is the disparity value, *d_min_* and *d_max_* are the shortest and longest depth values, which are chosen manually, and finally, *p_r_*, *p_g_*, and *p_b_* are the colorized pixels.

**Figure 3:**
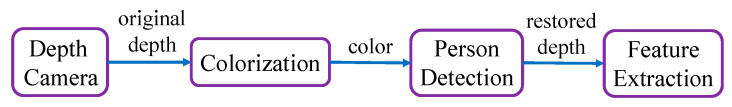
Alternating pixel values between depth and color values.

**Figure 4:**
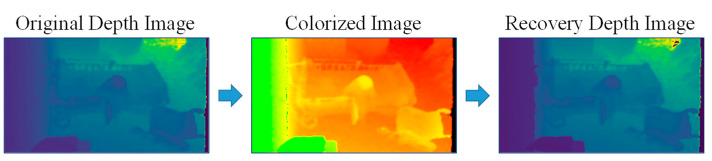
Results of the colorization process.

Although color images from the colorization are used to visualize, save, and detect a person, depth features are employed for the feature extraction process. Consequently, after completing the person detection process, the depth values (distance values) of the detected person have to be restored from the colorized images (color values). For this purpose, we calculate depth image recovery from the colorized images to extract depth features, using (6) and (7) from the same whitepaper [19]. The procedure for alternating pixel values between depth and color is shown in Figure 3.

(6) $$d_{rnormal}=\left\{\begin{array}{ll} p_{rg}-p_{rb}, & \;\mathrm{if}\;p_{rr}\geq p_{rg}\cap p_{rr}\geq p_{rb}\cap p_{rg}\geq p_{rb}\\ p_{rg}-p_{rb}+1529, & \;\mathrm{if}\;p_{rr}\geq p_{rg}\cap p_{rr}\geq p_{rb}\cap p_{rg}<p_{rb}\\ p_{rb}-p_{rr}+510, & \;\mathrm{if}\;p_{rg}\geq p_{rr}\cap p_{rg}\geq p_{rb}\\ p_{rr}-p_{rg}+1020, & \;\mathrm{if}\;p_{rb}\geq p_{rg}\cap p_{rb}\geq p_{rr} \end{array}\right.$$

(7) $$d_{recovery}=\frac{1529}{1529disp_{min}+\left(disp_{max}-disp_{min}\right)d_{rnormal}}$$

where *p_rr_*, *p_rg_*, and *p_rb_* are the pixels of the colorized depth image, *d_rnormal_* is the recovered normal depth value and *d_recovery_* is the restored depth value. The results of colorization from the depth image and the recovery from the colorized image are shown in Figure 4. The depth images are color-mapped images converted from depth values that are not suitable for visualization. The mean squared error is also calculated to compare the original depth values and recovery depth values. The equation for finding the mean squared error is described in (8).

(8) $$MSE=\frac{1}{mn}\sum_{i=0}^{m-1}\sum_{j=0}^{n-1}{\left[I\left(i,j\right)-K\left(i,j\right)\right]}^{2}$$

where *MSE* is the mean squared error between the original depth image *I* and the recovery depth image *K* for all pixels (*i, j*) in the image: the lower the *MSE* error, the greater the similarity between the two images. Our experiment determined that the average *MSE* error for 100 image pairs is 0.05.

### 3.2. Person Detection

The deep learning object detector YOLOv5 is used next for person detection. This algorithm is popular for having the best trade-off between speed and accuracy, in this case, 48.2% AP on the Common Objects in Context (COCO) dataset at 13.7 milliseconds [43,44]. Of the four types of YOLOv5 (YOLOv5s, YOLOv5m, YOLOv5l, and YOLOv5x, here ordered from smallest to largest), the YOLOv5l model is used as it performs better on our system. The colorized images are used as the input images for YOLOv5l.

For data preparation, the colorized images from Room 1 and Room 3 are used as a training and validation dataset, and images from Room 2 and Room 3 are used as a testing dataset. It should be noted that the training data used from Room 3 and the testing data used from Room 3 are not the same. Data from two rooms are used for the training dataset because these two rooms have differing structures and environments. Then, the images recorded in Room 1 and Room 3 are divided into an 80% training set and a 20% validation set to calculate the model’s performance. The performance evaluation metrics we obtained after training were 0.995 mAP@0.5 and 0.924 mAP@0.5:0.95.

**Figure 5:**
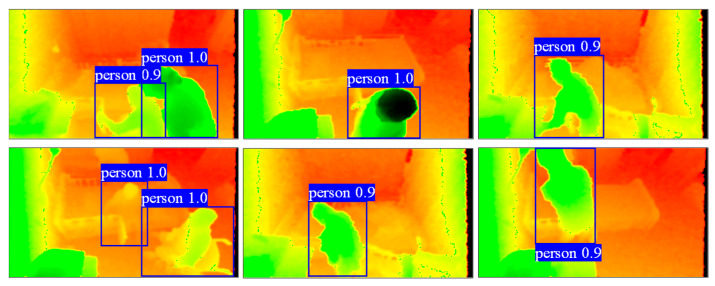
Sample person detection results.

After performing person detection with colorized images, the person’s bounding boxes which are automatically drawn around the person by the object detector, of various sizes, are obtained from the YOLOv5l inference. Figure 5 provides a sample of the results.

### 3.3. Feature Extraction

**Figure 6:**
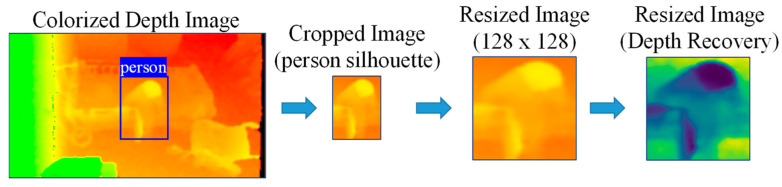
Person silhouette cropping, resizing, and depth recovery.

**Figure 7:**
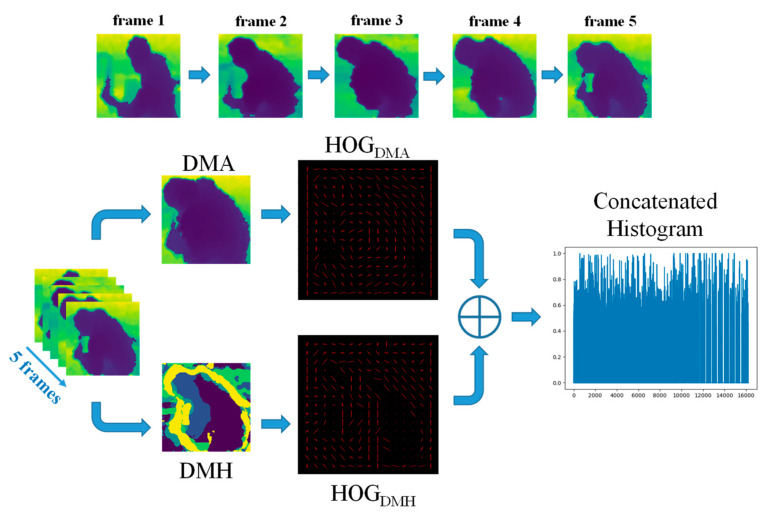
The architecture of feature extraction.

Before extracting features, the person bounding boxes resulting from the person detection process are cropped and then resized to a fixed size for normalization. We intend to extract appearance and motion depth map features from the depth image sequences. For this purpose, the depth recovery from the resized colorized image is calculated as an additional step as shown in Figure 6. The architecture of feature extraction is shown in Figure 7. Rather than using a single frame, an action sequence of five sequentially cropped person bounding boxes is used to extract features.

From this same action sequence, appearance features are then extracted using Depth Motion Appearance (DMA) by representing the overall shape and appearance, and motion features are extracted using Depth Motion History (DMH) by representing the temporal information for depth motion [13]. For instance, if the sequence has 60 frames, the feature extraction (DMA and DMH) will take place once every five frames from start to finish. Therefore, feature extraction occurs a dozen times through the 60 frames. To represent these appearance and motion features after each extraction from the action sequence, HOG is used as a descriptor. After that, HOGs from each feature are combined to form a concatenated histogram. Each HOG feature map is segmented into 8 × 8 pixels per cell, normalized using 2 × 2 blocks per cell, and then nine gradient directions are determined for each cell. As a result, each feature map had an 8100-dimensional HOG descriptor, resulting in a 16,200-dimensional concatenated histogram.

### 3.4. Action Recognition with HMM

This section defines each action recognized in our system, calculates HMM parameters and probability measures, and provides HMM prediction procedures.

#### 3.4.1. Action Definitions

**Figure 8:**
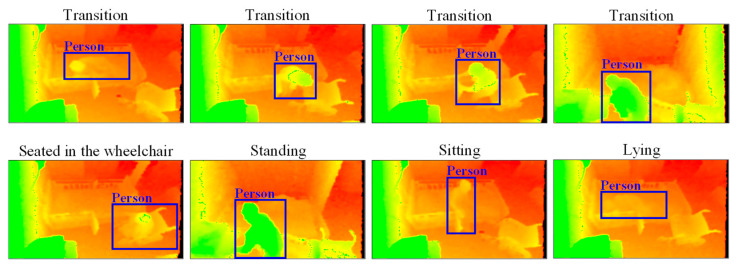
Specific action images.

This experiment is designed to recognize five actions: ‘Transition’, ‘Seated in the wheelchair’, ‘Standing’, ‘Sitting’, and ‘Lying’. Specific action images are shown in Figure 8. Of the five actions, ‘Transition’ is defined as the transitional state from one specific action to another. Though many transitional states occur from one activity to another, they all are combined and labeled as ‘Transition’ actions.

For each action, depth map features are extracted from five sequential frames, and then represented using HOG. As HMM is used for action recognition, the HOG feature vectors are transformed into observation sequences, and then fed into the HMM to predict various action sequences.

#### 3.4.2. HMM Parameters and Probability Measures

**Figure 9:**
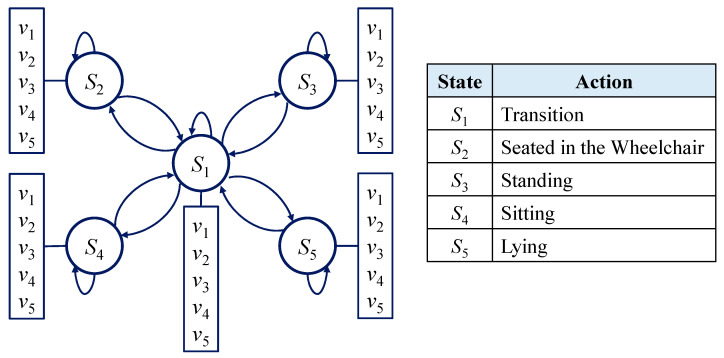
HMM model structure.

To characterize the HMM model, two model parameters and three probability measures are determined. The two model parameters are the number of states in the model, and the number of observation symbols per state, respectively. As there are five actions to be recognized and each action can be observed by a specific HOG feature, the states in the model are set as *S* = {*S*_1_, *S*_2_, *S*_3_, *S*_4_, *S*_5_}, and the five observation symbols per state are set as *V* = {*v*_1_, *v*_2_, *v*_3_, *v*_4_, *v*_5_}, which will be defined in (14) to (16) in Algorithm 1. The HMM model structure utilized for our system is shown in Figure 9. The three probability measures are the state transition probability distribution *A*, the emission probability distribution *B*, and the initial state distribution *π*. They can be described by the following equations.

(9) $$A=\{a_{ij}\},\;\;a_{ij}=P[q_{t}=S_{j}|q_{t-1}=S_{i}]$$

(10) $$B=\{b_{j}(k)\},\;\;b_{j}(k)=P[O_{t}=v_{k}|q_{t}=S_{j}]$$

(11) $$\pi =\{\pi_{i}\},\pi_{i}=P[q_{1}=S_{i}]\;\forall \;\;\;1\leq i,j,k\leq 5$$

(12) λ=(A,B,π)

where *a_ij_* is the transition from one state to another, *q_t_* is the state at time *t*, *b_j_*(*k*) is the occurrence probability in each state *j*, *O_t_* is the observation symbol at time *t*, *π_i_* is the initial probability for each state *i*, and *λ* is the complete HMM model.

Regarding the initial state distribution, we assume that equal probabilities apply for each action as in (13), below.

(13) $$\pi =\left[\begin{array}{ccccc} 0.2 & 0.2 & 0.2 & 0.2 & 0.2 \end{array}\right]$$

The state transition probability distribution *A* is calculated from a long-duration training sequence by creating the co-occurrence matrix of transitions from action to action ((*i, j*) pairs). 

To calculate emission probability distribution *B*, two training datasets that have an equal number of sample sequences for each action are used and they are shown in Table 2. The HOG features are extracted for each sequence of two datasets and each sequence has five continuous frames. The procedures are also explained in Algorithm 1.

**Figure 10:**
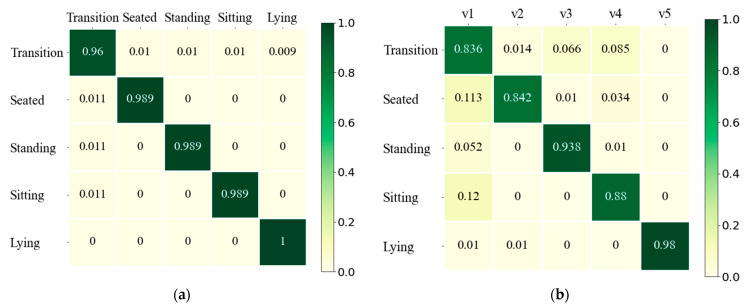
Heatmap visualization after training with Baum-Welch Algorithm for: (**a**) HMM transition probability matrix *A*; (**b**) HMM emission probability matrix *B*.

The calculated *A* and *B* are trained using the Baum-Welch Algorithm [45]. For the training process, the HOG features calculated from the sequences of Dataset-*Y* are transformed into the observation sequences. The probability results of *A* and *B* after the training process are shown in Figure 10, both of which provide heatmap visualizations in which the summation of probabilities for each row is one.
**Algorithm 1.** Calculation of *B* by Computing Mean HOGs**Input:** Dataset-*X*, Dataset-*Y***Output:** *B*: emission probability distribution1. **Function** MeanHOG_HMM (Dataset-*X*, Dataset-*Y*):2.  Calculate mean HOG feature vectors for each state of Dataset-*X* and set as *M*_1_*H*, *M*_2_*H*, *M*_3_*H*, *M*_4_*H*, and *M*_5_*H*.3.  Perform the following steps for each state of Dataset-*Y*.4.   Assign labels to all 100 input HOGs using (14) to (16).    $d(IH,MH)=\sqrt{\sum_{i=1}^{n}{\left(IH_{i}-MH_{i}\right)}^{2}},\;\;\;n=16200$    (14)    $k=\underset{1\leq j\leq 5}{\arg\min}[d(IH,M_{j}H)]$                (15)    $\mathrm{assign}\;\mathrm{label}=v_{k},\;\;\;\;\;k\in \left\{1,2,3,4,5\right\}$           (16)    Where *d*(*IH*, *MH*) is the Euclidean distance between input HOG *IH* and mean HOG *MH*, and *n* is the length of each HOG feature vector.5.   Calculate length (magnitude) of each labeled HOG.6.   Compute normal distribution for each HOG length.7.   Sum normal distributions which have the same labels.8.   Normalize five normal distributions by dividing each one with a summation of all normal distributions.9.  **Return** *B*10. **End Function**

After combining the *π* and the trained *A* and *B*, the required parameter set for the HMM model is obtained, and the Viterbi Algorithm is applied to find the hidden states.

#### 3.4.3. Procedures for HMM Prediction

**Figure 11:**
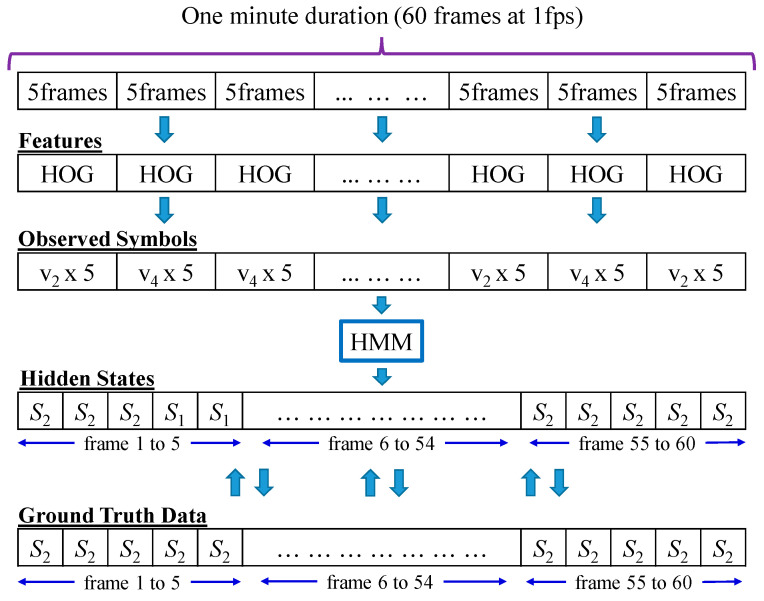
HMM, prediction on testing image sequences.

The HMM prediction for testing image sequences is shown in Figure 11, with a short sequence as a sample. This sample sequence has a duration of one minute, comprising 60 frames that we processed at a rate of 1fps. The HOG representations of depth appearance and motion features are extracted once every five frames, and the feature vectors are transformed as observed symbols. As one HOG provides one observation symbol, the observed symbol is duplicated to obtain five symbols each. After doing this, the 60 observed symbols that are the same as the input number of frames before extracting the HOG are obtained. These 60 observed symbols are then fed into the HMM for prediction using the Viterbi Algorithm, producing the hidden states. Each predicted hidden state is then compared one by one with ground truth data, and the accuracy is calculated according to the comparison results. For the example sequence in Figure 11, if 50 true predicted states exist in a total of 60 states, the accuracy will be calculated as 83.33%.

According to this HMM inferencing process, the accuracy of the HMM prediction (after applying the Viterbi Algorithm) tested on the training dataset (Dataset-*Y*) is 91%, and the confusion matrix is shown in Table 3.

#### 3.4.4. Alternative HMM Combinations

Our proposed system is also tested with two alternative HMM combinations, specifically by replacing some procedures in calculating the HMM model. The same transition probability matrix *A* and the initial distribution matrix *π* are used as in Section 3.4.2. However, some steps are changed in the procedures for calculating emission probability metric *B* in Algorithm 1. The mean HOG is calculated and the sequences from Dataset-*Y* are assigned labels according to the Euclidean distance between the input HOG and mean HOG in Algorithm 1. Instead of finding mean HOG and Euclidean distance, the alternative methods k-Nearest Neighbors (k-NN) and SVM are used, as described in Algorithms 2 and 3, respectively.

The Dataset-*X* and Dataset-*Y* used in these two alternative algorithms are the same as in Table 2. Using these two different emission probability matrices, the two HMM models are trained again using the Baum-Welch algorithm, which then performed the HMM predictions using the Viterbi Algorithm. According to testing on the training dataset (Dataset-*Y*), the accuracy of (k-NN + HMM) is 99%, that for (SVM + HMM) is also 99%, and their respective confusion matrices are shown in Table 4.

**Table 3:** Confusion matrix of HMM prediction results tested on the training dataset.

Mean + HMM					
Actual Actions	Predicted Actions				
	Transition	Seated	Standing	Sitting	Lying
Transition	90	1	7	2	0
Seated	14	86	0	0	0
Standing	7	0	93	0	0
Sitting	14	0	0	86	0
Lying	2	0	0	0	98

**Algorithm 2.** Calculation of *B* by Computing k-NN**Input:** Dataset-*X*, Dataset-*Y***Output:** *B*: emission probability distribution1. **Function** k-NN_HMM (Dataset-*X*, Dataset-*Y*):2.  Train the k-NN model using HOG features from Dataset-*X* and divide it into five classes such as *C*_1_, *C*_2_, *C*_3_, *C*_4_, and *C*_5_.3.  Perform the following steps for each state of Dataset-*Y*.4.   Assign labels to all 100 input HOGs using (17) and (18).      
k=kNNpred(IH)             (17)
     
$\mathrm{assign}\;\mathrm{label}=v_{k},\;\;\;\;\;k\in \left\{1,2,3,4,5\right\}$
   (18)     where *kNNpred*(*IH*) is k-NN prediction on HOGs.5.   Calculate length (magnitude) of each labeled HOG.6.   Compute normal distribution for each HOG length.7.   Sum normal distributions which have the same labels.8.   Normalize five normal distributions by dividing each one with a summation of all normal distributions.9.  **Return** *B*10. **End function**


**Algorithm 3.** Calculation of *B* by Computing SVM**Input:** Dataset-*X*, Dataset-*Y***Output:** *B*: emission probability distribution1. **Function** SVM_HMM (Dataset-*X*, Dataset-*Y*):2.  Train the SVM model using HOG features from Dataset-*X* and divide it into five classes such as *C*_1_, *C*_2_, *C*_3_, *C*_4_, and *C*_5_.3.  Perform the following steps for each state of Dataset-*Y*.4.   Assign labels to all 100 input HOGs using (19) and (20).      k=SVMpred(IH)               (19)      $\mathrm{assign}\;\mathrm{label}=v_{k},\;\;\;\;\;k\in \left\{1,2,3,4,5\right\}$    (20)      where *SVMpred*(*IH*) is *SVM* prediction on HOGs.5.   Calculate length (magnitude) of each labeled HOG.6.   Compute normal distribution for each HOG length.7.   Sum normal distributions which have the same labels.8.   Normalize five normal distributions by dividing each one with a summation of all normal distributions.9.  **Return** *B*10. **End function**


**Table 4:** Confusion matrices of HMM prediction results tested on the training dataset.

(a) k-NN + HMM					
Actual Actions	Predicted Actions				
	Transition	Seated	Standing	Sitting	Lying
Transition	100	0	0	0	0
Seated	1	99	0	0	0
Standing	0	0	100	0	0
Sitting	1	0	0	99	0
Lying	1	0	0	0	99
**(b) SVM + HMM**					
**Actual Actions**	**Predicted Actions**				
	**Transition**	**Seated**	**Standing**	**Sitting**	**Lying**
Transition	100	0	0	0	0
Seated	0	100	0	0	0
Standing	1	0	99	0	0
Sitting	2	0	0	98	0
Lying	0	0	0	0	100

## 4. Experimental Results

In this section, the performance of our system is analyzed using real-world data. Firstly, a comparison was made between three models that combined the HMM with a selection of classification algorithms. In addition, this section also compares the recognition accuracy of the proposed methods with those used in previous works.

### 4.1. Data Preparation

Our observations were made in three separate rooms, in each of which a senior person was residing. Although most of the data from Rooms 1 and 3 were used as a training set, sequences from all three rooms (Room 1, Room 2, Room 3) were used for testing purposes. All sequences were trimmed from the first frame in which a resident entered the room to the last frame when the subject left the room, and were recorded at 1fps.

### 4.2. Action Recognition and Result Comparison

From start to finish through each testing sequence, the HMM action recognition was performed once a minute. The results were then compared with ground truth action labels to calculate accuracy. Table 5 compares the three proposed methods. The average accuracy with each method in each room is shown, indicating that the combination of SVM classification with HMM in calculating the emission probability matrix achieved the highest accuracy at 84.04% for all testing sequences. Furthermore, Table 6 shows the recognition accuracy rate for each specific action, each tested using the SVM + HMM method in each testing room.

Again, Table 7 also shows the action recognition results of using the SVM + HMM method for each sequence in each room. The tables describe the recorded data from the three rooms, including the duration, number of frames, start date, and time for each sequence, as well as the processing time. The sequences are presented in order from the shortest to the longest duration. The results showed that the average accuracy for Room 1 sequences was 90.28%, and those for Rooms 2 and 3 were 81.37% and 80.48%, respectively. The testing process was performed using a 64-bit Intel (R) Core i9 with 64GB RAM and NVIDIA GeForce RTX 3090 GPU. The experimental results from Table 7 also show that our proposed system can provide real-time action recognition on the continuous, long-duration frame sequences.

Further comparison between the proposed HMM methods and related methods in previous works are shown in Table 8. For the sake of comparison, the different approaches to input data with different methods from the previous works were chosen. Since the recognized actions for each method are not the same, we included only the number of actions in the comparison table. The results show that our proposed approach and method performed better than the other methods in the recognition accuracy rate. Additionally, the proposed system can recognize actions at night without using additional light, which is not possible with most of the other approaches. Even more, our proposed system is inexpensive to set up because it does not require many ambient sensors inside the rooms or wearable sensors.

## 5. Discussion

In this paper, a stereo depth camera was used as an approach for recognizing actions performed by older people at a care center. Creating color images by colorizing depth maps greatly reduced the file sizes as compared with using CSV files, and enabled clearly visualizing depth maps. Moreover, the colorized images could be used as direct input for YOLOv5, and depth maps could not. The application of YOLOv5 person detection improved our process over that in previous work [13,14], which used simpler, traditional methods for person detection. These improved results for person detection improved results for action recognition in the proposed system. Regarding the action recognition process, we tried out three combinations of HMM with classification algorithms and found that the fusion of HMM with SVM outperformed other combinations in accuracy in continuous operations.

This study highlighted the fact that the proposed system could assist family members and caregivers, as it provides real-time action recognition. By using this system, caregivers do not need to visit the elderly residents as often, especially useful during the COVID-19 pandemic period. In daily use, the system will store an increasingly large amount of data over time, which will allow tracking changes in behavior patterns using these action histories. Analysis of the data suggests that the system can detect the ‘lying on the bed’ action and thus sleep quality can be evaluated and sleep profiles can be provided. These are important indicators of health, which can also be used to improve long-term care. Furthermore, this study indicates that the system could help healthcare specialists make timely decisions, by providing details and summarized actions. Since the proposed algorithm can detect the daily actions of the elderly, we believe that these findings would be beneficial to quality long-term care.

The findings of this study should be interpreted with caution in that the data are obtained from only three rooms from one care center. The system does not always travel well, as it produced some false positives when used to recognize actions in rooms that differ in structure or environment from those in the training rooms. Besides, the system still has some recognition challenges. As described in Table 6, it confused some actions with ‘Transition’ actions which have lower accuracy rates, such as in the following three situations:

1. Situation 1: Person is lying on the bed improperly,
2. Situation 2: Person cannot be seen clearly,
3. Situation 3: Two activities have similar appearance and motion patterns.

**Figure 12:**
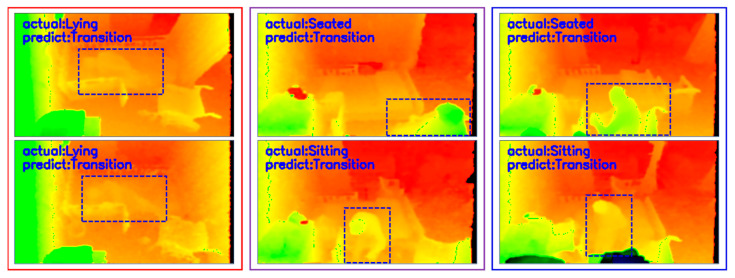
Examples of the common false action recognitions in Situation 1 (**left** two images); Situation 2 (**middle** two images); and Situation 3 (**right** two images).

Some examples of common false recognition in these three situations are shown in Figure 12. Another interesting area for further research would be to examine the ‘Transition’ action in more detail and analyze the high-risk transitional states from one action to another.

There are a bunch of useful assistive systems such as the fall risk assessment system [46], ambient long-term gait assessment system [47], and radar-based real-time action recognition system [48], which were invented in recent years to prevent accidents and abnormal events. Since the proposed system can monitor the elderly for 24 h a day in real-time and recognize their actions independently, it is obvious that our proposed system can also be utilized as an assistive technology system for not only the elderly but also their caregivers.

## 6. Conclusions

In this paper, we have established a system with the cooperation of elderly people, who willingly participated partly because the system does not require to put wearable sensors. In addition, the depth camera used in the system not only protects the privacy of the elderly, but can also be used at night. By using this action recognition system, we can not only reduce the workload for caregivers and lower costs, but also provide them with useful and insightful information.

As for future research, we would like to remove the colorization process from the system framework, using only depth data (distance data) obtained directly from the camera. We would also like to use the action recognition histories in analyzing behavior and sleep quality. Furthermore, the development of e-Healthcare (electronic healthcare) systems is expected in the coming years, which will rely on the application of information and communication technologies. Hopefully, our proposed system will enable transformational improvements in elder care.

## Figures and Tables

**Table 1:** Comparison of system storage space.

Data Type	Data Recorded Duration	Storage Space
CSV files	1 h	1.73 GB
Colorized Images	1 h	100 MB

**Table 2:** Training datasets used for calculating HMM emission probability distribution *B*.

Action	Transition	Seated	Standing	Sitting	Lying
State	*S*_1_	*S*_2_	*S*_3_	*S*_4_	*S*_5_
# Sequence in Dataset-*X*	900	900	900	900	900
# Sequence in Dataset-*Y*	100	100	100	100	100

**Table 5:** Comparison of three methods.

Room ID	Total Sequences	Average Accuracy for All Sequences after Testing with Three Methods (%)		
		Mean + HMM	k-NN + HMM	SVM + HMM
1	22	87.05	95.19	90.28
2	10	74.83	89.01	81.37
3	17	79.41	57.56	80.48
Average Accuracy		80.43	80.59	84.04

**Table 6:** Accuracy for each specific action tested with the SVM + HMM method.

Room ID	Accuracy (%)					
	Transition	Seated	Standing	Sitting	Lying	Overall
1	63.37	95.45	97.24	95.41	91.65	90.28
2	54.93	75.09	-	98.83	74.61	81.37
3	74.13	48.68	59.96	83.87	91.89	80.48
Average	64.14	73.07	78.60	92.70	86.05	84.04

**Table 7:** Accuracy for each testing sequence from each room tested with the SVM + HMM method.

(a) Room 1 Sequences					
Sequence	Duration (hour)	Total Frames (1fps)	Start Date and Time	Accuracy (%)	Processing Time (hour)
Seq_1	0.05	168	21 October 2019_07:19:43	86.31	0.02
Seq_2	0.12	433	24 October 2019_08:44:51	87.76	0.07
Seq_3	0.15	530	21 October 2019_18:25:28	85.66	0.07
Seq_4	0.34	1230	19 October 2019_12:47:07	72.11	0.19
Seq_5	0.47	1684	21 October 2019_17:37:38	91.75	0.25
Seq_6	0.61	2192	22 October 2019_17:16:55	94.30	0.33
Seq_7	0.62	2235	19 October 2019_13:32:27	96.06	0.35
Seq_8	1.08	3876	24 October 2019_07:27:36	83.18	0.58
Seq_9	1.85	6673	24 October 2019_11:34:20	86.68	1.00
Seq_10	1.86	6687	19 October 2019_08:12:33	95.10	1.02
Seq_11	2.60	9347	21 October 2019_11:54:52	90.46	1.38
Seq_12	2.74	9865	20 October 2019_11:44:59	97.77	1.51
Seq_13	2.80	10,063	22 October 2019_11:19:33	85.44	1.55
Seq_14	3.04	10,927	18 October 2019_12:08:33	99.35	1.66
Seq_15	3.25	11,688	23 October 2019_11:38:46	96.89	1.78
Seq_16	7.34	26,412	12 October 2019_20:54:48	96.41	4.05
Seq_17	10.98	39,535	20 October 2019_18:57:47	86.78	6.10
Seq_18	11.16	40,158	21 October 2019_18:40:47	86.38	6.23
Seq_19	11.50	41,407	22 October 2019_18:02:55	92.67	6.42
Seq_20	11.73	42,234	18 October 2019_17:58:57	91.69	6.90
Seq_21	12.18	43,842	23 October 2019_17:05:09	93.37	7.36
Seq_22	12.60	45,366	19 October 2019_17:51:09	90.11	7.99
Average Accuracy				90.28	
**(b) Room 2 Sequences**					
**Sequence**	**Duration** **(hour)**	**Total Frames** **(1fps)**	**Start Date and Time**	**Accuracy** **(%)**	**Processing Time (hour)**
Seq_1	0.10	362	26 October 2019_07:20:50	67.96	0.05
Seq_2	0.30	1078	26 October 2019_07:32:06	92.12	0.18
Seq_3	0.68	2455	28 October 2019_04:45:41	93.93	0.4
Seq_4	1.17	4214	26 October 2019_06:02:33	92.05	0.71
Seq_5	2.17	7808	27 October 2019_04:35:50	83.03	1.32
Seq_6	2.36	8494	26 October 2019_09:54:09	94.59	1.43
Seq_7	2.64	9488	25 October 2019_11:07:16	77.18	1.58
Seq_8	11.35	40,846	25 October 2019_17:01:02	53.04	6.89
Seq_9	11.73	42,214	26 October 2019_15:10:58	63.57	7.55
Seq_10	12.19	43,896	27 October 2019_14:02:05	96.22	7.4
Average Accuracy				81.37	
**(c) Room 3 Sequences**					
**Sequence**	**Duration** **(hour)**	**Total Frames** **(1fps)**	**Start Date and Time**	**Accuracy** **(%)**	**Processing Time (hour)**
Seq_1	0.05	171	26 October 2019_07:56:27	84.80	0.03
Seq_2	0.42	1511	27 October 2019_04:24:47	72.34	0.27
Seq_3	0.45	1630	12 October 2019_12:09:13	93.56	0.28
Seq_4	0.54	1941	26 October 2019_07:23:18	80.94	0.35
Seq_5	0.65	2325	22 October 2019_19:16:20	81.29	0.38
Seq_6	0.87	3146	25 October 2019_12:21:40	69.52	0.55
Seq_7	1.03	3697	12 October 2019_13:47:28	93.70	0.64
Seq_8	1.42	5112	27 October 2019_06:12:16	64.10	0.91
Seq_9	2.01	7240	28 October 2019_05:11:31	79.93	1.29
Seq_10	10.58	38,085	18 October 2019_19:45:54	79.95	6.37
Seq_11	10.71	38,551	20 October 2019_19:23:14	77.13	6.57
Seq_12	11.28	40,608	12 October 2019_17:54:12	93.84	6.83
Seq_13	11.38	40,971	26 October 2019_16:48:13	71.05	5.85
Seq_14	11.69	42,092	19 October 2019_19:33:25	73.67	7.31
Seq_15	11.74	42,263	21 October 2019_18:32:10	70.23	7.42
Seq_16	11.90	42,823	27 October 2019_15:47:34	92.31	7.18
Seq_17	11.91	42,882	25 October 2019_17:39:06	89.82	7.38
Average Accuracy				80.48	

**Table 8:** Comparison of recognition accuracy between the proposed methods and those in previous works.

Approach	Method	No. of Actions	Accuracy (%)
RGB Images	CNN [10]	15	71.00
Skeleton	Random Forest [15]	20	70.00
Sensor Data	Two-Layer HMM [34]	13	74.85
Sensor Data	Hierarchical HMM [35]	12	65.20
Depth Images	Mean HOG + HMM (Proposed)	5	80.43
Depth Images	kNN + HMM (Proposed)	5	80.59
Depth Images	SVM + HMM (Proposed)	5	84.04

## Data Availability

The data presented in this study are available on request from the corresponding author.
